# Insights into the genetic variation of maternal behavior and suckling performance of continental beef cows

**DOI:** 10.1186/s12711-016-0223-z

**Published:** 2016-06-22

**Authors:** Alexis Michenet, Romain Saintilan, Eric Venot, Florence Phocas

**Affiliations:** GABI, INRA, AgroParisTech, Université Paris-Saclay, 78350 Jouy-en-Josas, France; AURIVA, Les Nauzes, 81580 Soual, France; ALLICE, 149 rue de Bercy, 75012 Paris, France

## Abstract

**Background:**

In beef cattle, maternal care is critical for calf survival and growth. Our objective was to evaluate the major sources of additive genetic variation in maternal behavior and suckling performance in two genetically close beef breeds.

**Methods:**

Maternal performance was assessed based on maternal behavior (MB), milk yield (MY) and udder swelling score (US) of 1236 Blonde d’Aquitaine cows and 1048 Limousin cows. MB was scored just after calving to describe the intensity of the dam’s protective behavior towards her calf. Most of the cows were genotyped using the low-density chip EuroG10K BeadChip, and imputed to the high-density 770K panel within breed. Genetic parameters for each trait were estimated for each breed under a multi-trait best linear unbiased prediction animal model. Genomic analysis was performed for each breed using the high-density genotypes and a Bayesian variable selection method.

**Results:**

Heritabilities were low for MB (0.11–0.13), intermediate for MY (0.33–0.45) and high for US (0.47–0.64). Genetic correlations between the traits ranged from 0.31 to 0.58 and 0.72 to 0.99 for the Blonde d’Aquitaine and Limousin breeds, respectively. Two quantitative trait loci (QTL) were detected for MB in Blonde d’Aquitaine with *NPY1R* and *ADRA2A* as candidate causative genes. Thirty to 56 QTL were detected for MY and US in both breeds and 12 candidate genes were identified as having a role in the genetic variation of suckling performance. Since very few pleiotropic QTL were detected, there was little biological explanation for the moderate (0.57) to very high (0.99) genetic correlations estimated between MY and US in the Blonde d’Aquitaine and Limousin cows, respectively. In Blonde d’Aquitaine, the correlation was largely due to the pleiotropic QTL detected in the region upstream of the *CG* gene, while in Limousin, this region was only identified for US, thus attesting the difference in genetic architecture between the breeds.

**Conclusions:**

Our findings question the assumption that two populations that have close genetic links share many QTL. Nevertheless, we identified four candidate genes that may explain a substantial amount of the genetic variation in suckling performance of these two breeds.

**Electronic supplementary material:**

The online version of this article (doi:10.1186/s12711-016-0223-z) contains supplementary material, which is available to authorized users.

## Background

Developments in molecular biology and statistical methodologies have provided new tools to unravel the genetics of complex traits in farm animals. Maternal traits in cattle are of particular economic importance to breeders, but because performance records for these traits are limited in the beef sector, there is a lack of efficient selection tools to improve such traits. Traits related to the udder and milk production influence calf growth and weaning weight, which have a direct effect on the income of livestock farmers [1]. Creation of the maternal bond generates diverse behaviors in cows such as sniffing, licking and protecting the neonate to ensure the growth and survival of their calves [2]. It has been reported that nulliparous dams immediately before or during fetal delivery develop an intense interest for the neonate, due to the action of several hormones [3], some of which (such as oxytocin) are also linked to milk release after teat stimulation by suckling [4]. Therefore, traits that affect maternal care and milk production may be genetically linked. In addition, the maternal behavior of the dam impacts calf survival [5] which is one of the main traits that affects the economic performance of suckler production systems [6]. The aim of this study was to increase knowledge about the major sources of additive genetic variance for some important maternal traits in beef cows that are linked to their behavior and milk production. Rare phenotypes for primiparous beef cows were available for this study: udder swelling before calving, maternal behavior after calving, and milk production. These traits were recorded in two distinct but genetically close breeds [7]: Blonde d’Aquitaine and Limousin. These two breeds are relatively close since the Blonde d’Aquitaine breed was granted an official new breed status in 1962 by associating three branches of this breed from the south-west of France: Garonnaise, Pyrénéenne and Blonde du Quercy (the latter being very similar to Limousin) [8].

The primary objective of our study was to validate the existence of strong genetic correlations between the three maternal traits mentioned above in these two breeds, based on the classic infinitesimal polygenic model. The second aim was to identify the main chromosomal regions that play a role in each of the three maternal traits within the breeds, and to identify putative candidate genes that might have a pleiotropic effect on these traits. The third aim was to compare the results between the breeds in order to determine whether the same quantitative trait loci (QTL) for maternal traits segregate in these two genetically close cattle populations.

## Methods

Approval from the Animal Care and Use Committee was not required for this study because the data were collected routinely as part of the breeding program and the determination of phenotypes did not violate the integrity of the animals.

### Animals and phenotypes

Data were collected from the French progeny-testing programs for artificial insemination (AI) using bulls from the Blonde d’Aquitaine and Limousin breeds. Three traits that are linked to maternal care were recorded on 2284 purebred females that were born between 2002 and 2011 and were the daughters of 70 Blonde d’Aquitaine sires and 57 Limousin sires. The number of records, raw means and raw variances for each trait are in Table 1.

**Table 1:** Number of records, mean and variance of traits for Blonde d’Aquitaine and Limousin primiparous cows

	Blonde d’Aquitaine			Limousin		
Trait	US	MB	MY	US	MB	MY
Number of records	1236	1212	1127	1048	1048	1036
Mean	3.01	4.45	5.54	3.64	4.14	6.61
Variance	0.75	1.42	1.91	0.82	0.63	3.13

*MB* Maternal Behavior, *US* Udder Score, *MY* Milk Yield

The udder score (US) that assesses udder swelling was scored 1 week before calving according to a five-point scale ranging from 1 for the least swollen udder to 5 for the most swollen udder (see Additional file 1). Maternal behavior (MB) was scored during the first hour after parturition and it aimed at describing the intensity of the dam’s protective behavior towards her calf. A score of 1 was assigned to a dam that did not pay attention to her calf, while a score of 5 was assigned to a dam that actively stimulated the newborn calf to suck, particularly by licking it immediately after calving. Milk yield (MY) was assessed using the calf weigh-suckle-weigh technique to estimate milk yield by calculating the difference in calf weights before and after suckling (see Additional file 1).

### Genotypes

In total, 2269 females (1259 Limousin and 1010 Blonde d’Aquitaine cows) were genotyped. Most of the females were genotyped using the customized low-density chip EuroG10K BeadChip^®^. Only 249 Blonde d’Aquitaine cows were genotyped with the Bovine SNP50 BeadChip^®^ [medium-density (MD) chip]. Most of the sires (69 of 78 Blonde d’Aquitaine and 39 of 57 Limousin sires) were genotyped using the Bovine HD BeadChip^®^ corresponding to a high-density (HD) chip with 777K single nucleotide polymorphisms (SNPs). The remaining sires were genotyped with the MD chip. All female genotypes were imputed to HD by applying a two-step procedure, from low-density to MD, then from MD to HD, using the BEAGLE 3.3.0 software [9]. The procedures used for genotype editing and imputation are described in Hozé et al. [10]. Error rates of allelic imputation from low-density to MD were estimated at 1.3 % for Blonde d’Aquitaine and 1.6 % for Limousin [11] and those from MD to HD were estimated at 1.8 and 1.1 %, respectively [10]. After editing, 706,791 SNPs were used for the analysis. SNPs were mapped to the UMD 3.1 bovine genome sequence assembled by the Center of Bioinformatics and Computational Biology at the University of Maryland.

### Statistical model used to estimate genetic parameters

The analyses were performed using a multi-trait linear model, considering the animal genetic effect for each trait i:

$${\mathbf{y}}_{\text{i}} = {\mathbf{X}}_{\text{i}} {\varvec{\upbeta}}_{\text{i}} + {\mathbf{Z}}_{\text{i}} {\mathbf{a}}_{\text{i}} + {\mathbf{e}}_{\text{i}} ,$$

where ${\mathbf{y}}_{\text{i}}$ is the vector of observations, ${\varvec{\upbeta}}_{\text{i}}$ is the vector of fixed effects, ${\mathbf{a}}_{\text{i}}$ is the vector of random effects of cow, ${\mathbf{e}}_{\text{i}}$ is the vector of random residual effects, and ${\mathbf{X}}_{\text{i}}$, and ${\mathbf{Z}}_{\text{i}}$ are the corresponding incidence matrices for trait i. It is assumed that ${\text{E}}\left[ {{\mathbf{y}}_{\text{i}} } \right] = {\mathbf{X}}_{\text{i}} {\varvec{\upbeta}}_{\text{i}}$, $${\text{E}}\left[ {\begin{array}{*{20}c} {{\mathbf{a}}_{\text{i}} } \\ {{\varvec{\upvarepsilon}}_{\text{i}} } \\ \end{array} } \right] = \left[ {\begin{array}{*{20}c} 0 \\ 0 \\ \end{array} } \right]$$ and $${\text{Var}}\left[ {\begin{array}{*{20}c} {{\mathbf{a}}_{\text{i}} } \\ {{\varvec{\upvarepsilon}}_{\text{i}} } \\ \end{array} } \right] = \left[ {\begin{array}{*{20}c} {{\mathbf{A}}\upsigma_{{{\text{a}}_{\text{i}} }}^{2} } & 0 \\ 0 & {{\mathbf{I}}\upsigma_{{\upvarepsilon_{\text{i}} }}^{2} } \\ \end{array} } \right]$$. Noting $${\mathbf{a}} = \left[ {\begin{array}{*{20}c} {{\text{a}}_{1} } \\ {{\text{a}}_{2} } \\ {{\text{a}}_{3} } \\ \end{array} } \right]$$ and $${\mathbf{e}} = \left[ {\begin{array}{*{20}c} {{\text{e}}_{1} } \\ {{\text{e}}_{2} } \\ {{\text{e}}_{3} } \\ \end{array} } \right]$$, the genetic ${\mathbf{G}}$ and residual ${\mathbf{R}}$ variance–covariance matrices for the multi-trait model were defined as follows:

$${\mathbf{G}} = {\mathbf{A}} \otimes \left[ {\begin{array}{*{20}c} {\sigma_{{a_{1} }}^{2} } & {\sigma_{{a_{1} a_{2} }} } & {\sigma_{{a_{1} a_{3} }} } \\ {\sigma_{{a_{2} a_{1} }} } & {\sigma_{{a_{2} }}^{2} } & {\sigma_{{a_{2} a_{3} }} } \\ {\sigma_{{a_{3} a_{1} }} } & {\sigma_{{a_{3} a_{2} }} } & {\sigma_{{a_{3} }}^{2} } \\ \end{array} } \right]$$

and

$${\mathbf{R}} = {\mathbf{I}} \otimes \left[ {\begin{array}{*{20}c} {\sigma_{{e_{1} }}^{2} } & {\sigma_{{e_{1} e_{2} }} } & {\sigma_{{e_{1} e_{3} }} } \\ {\sigma_{{e_{2} e_{1} }} } & {\sigma_{{e_{2} }}^{2} } & {\sigma_{{e_{2} e_{3} }} } \\ {\sigma_{{e_{3} e_{1} }} } & {\sigma_{{e_{3} e_{2} }} } & {\sigma_{{e_{3} }}^{2} } \\ \end{array} } \right],$$

where $\sigma_{{a_{i} }}^{2}$ is the additive genetic variance for trait ${\text{i}}$, $\sigma_{{a_{i} a_{j} }}$ is the genetic covariance between traits ${\text{i}}$ and ${\text{j}}$, $\sigma_{{\varepsilon_{i} }}^{2}$ is the residual variance for trait ${\text{i}}$, $\sigma_{{a_{i} a_{j} }}$ is the residual covariance between traits ${\text{i}}$ and ${\text{j}}$, ${\mathbf{A}}$ is the relationship matrix, and ${\mathbf{I}}$ is the identity matrix.

The relationship matrices included 6657 Blonde d’Aquitaine and 8836 Limousin animals with pedigrees that could be traced back five generations. The discrete nature of the US and MB scores was not taken into account because linear models can perform evenly or better than threshold models when assessing genetic values for which several ordinal categories are considered or the amount of information per level of fixed effect is small [12, 13]. Moreover, estimation of genetic correlations is not affected by the statistical treatment (linear or threshold model) of the categorical trait [14].

The fixed effects considered for the three traits were the birth region in France (six and five levels for the Blonde d’Aquitaine and Limousin breeds, respectively), birth year-season of the heifers (four seasons), gender of the calf and effect of the mating bull (two bulls per breed). The age at calving (in days) was also fitted as a co-variable for all traits. With respect to the US and MB traits, the calving period (three levels) within the year was added to the previous model. For MY, the suckling batch (four and six levels for the Blonde d’Aquitaine and Limousin breeds, respectively) within 1 year and the calving difficulty score (two levels: easy calving versus hard pull or caesarean section) were added to the model.

Analyses were performed using WOMBAT software [15].

### Statistical approach for the detection of QTL

Because the number of SNP effects that had to be estimated was larger than the number of records, detection of QTL was based on a Bayesian variable selection approach [16]. We considered a BayesC strategy [17] where a fraction of the SNPs, $\uppi$, was assumed to have a non-zero effect at each iteration. SNP effects were estimated using a mixture of a proportion $\uppi$ of SNPs with a normal effect distribution ${\text{N(}}0,\upsigma_{a}^{2} )$ and a proportion $1 -\uppi$ of SNPs with mass point distribution at 0. The general linear mixture model was defined as follows:

$$y_{i} = \upmu + \mathop \sum \limits_{j = 1}^{n} z_{ij} a_{j} \delta_{j} + e_{i} ,$$

where $y_{\text{i}}$ is the phenotype adjusted for environmental effects of animal ${\text{i}}$, $\upmu$ the mean for the trait considered, $n$ the number of SNPs, $z_{\text{ij}}$ the genotype at locus ${\text{j}}$ for animal ${\text{i}}$ (with $z_{\text{ij}} = 1$ for the homozygote with allele 1 at locus ${\text{j}}$, $z_{\text{ij}} = - 1$ for the opposite heterozygote and $z_{\text{ij}} = 0$ for the heterozygote), $a_{\text{j}}$ the effect for SNP ${\text{j}}$, $\delta_{\text{j}}$ the indicator variable ($\delta_{\text{j}} = 1$ if SNP ${\text{j}}$ was selected at a given iteration, $\delta_{\text{j}} = 0$, otherwise), and $e_{\text{i}}$ the random residual effect. We used GS3 software [18] to perform these analyses. A total of 100,000 iterations were performed, with a burn-in of 20,000 iterations. The retained $\uppi$ value was equal to 0.025 %, corresponding to 177 SNPs selected at each iteration among all the SNPs on the HD chip, in order to have a smaller number of SNP effects to estimate at each iteration than the number of phenotypic records. This value of $\uppi$ was sufficient to completely capture the additive variance derived under the pedigree BLUP animal model. We verified that adding a polygenic component to the sum of the SNP effects in the model did not help to capture any spurious associations and did not modify the results of the pure genomic model. Therefore, a pure genomic model was retained to present the results.

#### Definition of the QTL regions

The degree of association between each SNP and the phenotypes was assessed using the Bayes factor (BF) [19]. This involves $\uppi$ and ${\text{P}}_{\text{i}}$ as in the following equation [20], ${\text{P}}_{\text{i}}$ being the probability for the SNP of having a non-zero effect:

$${\text{BF}} = \frac{{\text{P}}_{\text{i}} /{( {1 - {\text{P}}_{\text{i}}})}}{\uppi /{( {1 -\uppi})}}$$

The Bayes factor offers a rigorous and clear framework to compare competing models. It is the recommended statistical criterion when using the Bayesian method to detect QTL [21, 22].

**Fig. 1:**
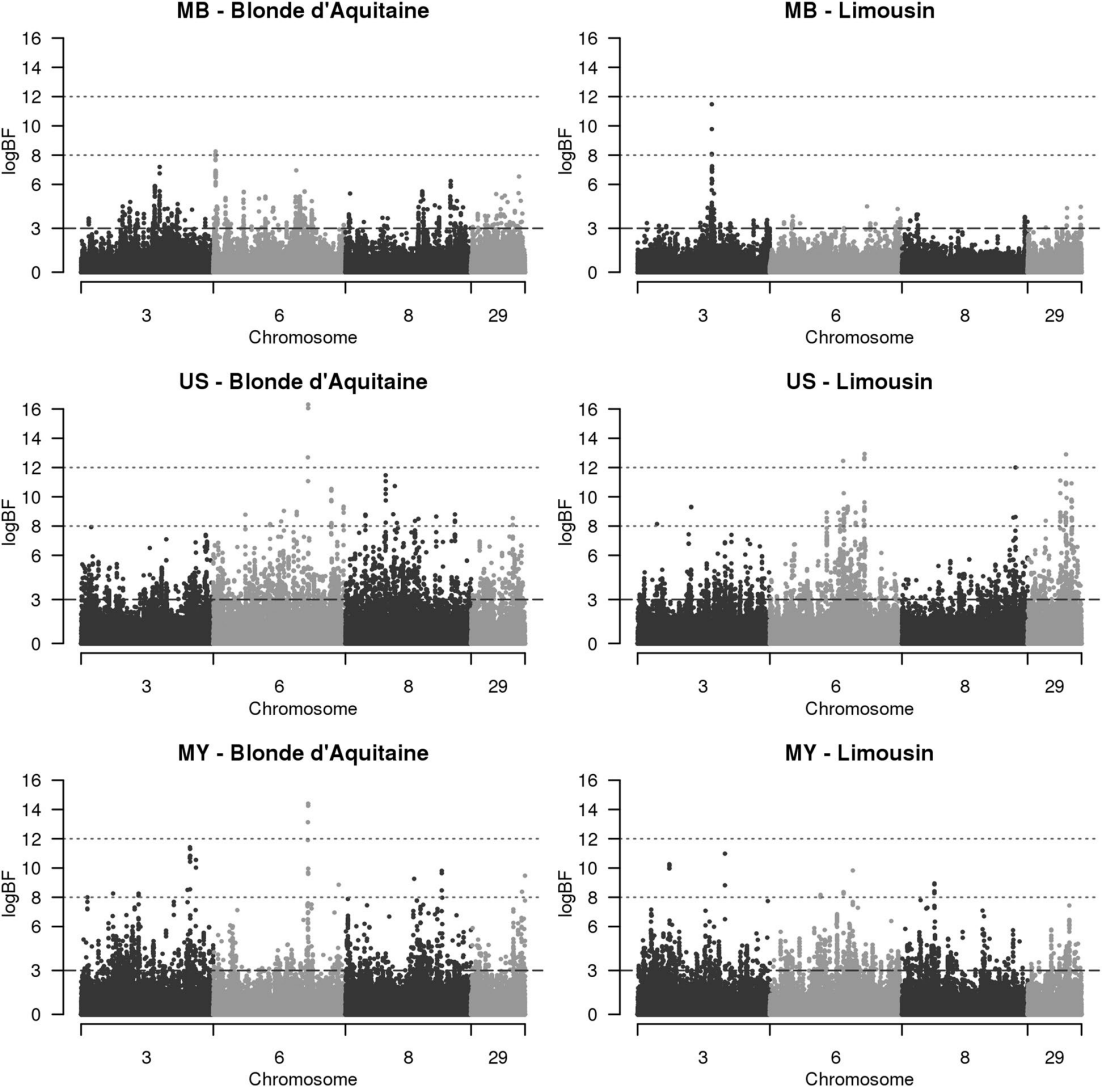
Chromosome plots of logBF for maternal behavior, udder swelling score and milk yield for the two breeds. The logBF values for all SNPs were plotted for chromosomes 3, 6, 8, and 29 for the detection of QTL for maternal behavior (MB), udder swelling score (US) and milk yield (MY)

A transformation of BF (logBF), which is computed as twice the natural logarithm of the BF, was considered in order to obtain a clearer visual appraisal of all QTL regions at the scale of the chromosome (see Fig. 1). Since this logarithmic scale produced values within the same usual range as the deviance and likelihood ratio test values, it facilitated the determination of thresholds to define QTL as proposed by Kass and Raftery [20]. Indeed, these authors suggested the following categories to classify the strength of the evidence provided by logBF: evidence in favor of the existence of the effect is positive for values within the interval (3, 6], strong for values within the interval (6, 10] and very strong for values higher than 10. In this study, we assumed that a chromosomal region qualified as a QTL when it contained at least one SNP with a peak logBF above a threshold of 8 (BF ≈ 55). We considered that the evidence for this QTL was strong when the peak logBF was above a threshold of 12 (BF ≈ 400). Finally, we considered a chromosomal region as a “putative QTL” when it contained at least one SNP with a logBF value within the interval (6, 8[.

Accuracy of genomic prediction in the reference population was estimated by calculating the correlation coefficient r between the phenotypes and the genomic predicted breeding values that were derived from the effects of the 706,791 SNPs. This estimated accuracy is a proxy for the true accuracy of genomic prediction which is the correlation between the genomic predicted breeding values and the true breeding values (instead of the phenotypes). The contribution of each QTL in the total genomic prediction was assessed by considering the ratio of the correlation between the phenotypes and the partial genomic values derived from the sum of the effects of SNPs within the QTL region over the total accuracy r. This ratio aims at quantifying the impact of the QTL on the estimated accuracy of genomic prediction for each trait.

Because SNPs with the highest logBF are not necessarily those that are closest to the causal mutation, SNPs that had a logBF higher than 3 and were located close to the peak SNP were also included in the QTL region when these SNPs were within a sliding window of 0.5 Mb on each side of the peak SNP. The sliding window approach was applied when at least one SNP with a logBF higher than 3 was found in the current window. The start and end bounds of the QTL regions were defined by the locations of the last SNP that had a logBF higher than 3 and was within the QTL region.

## Results and discussion

### Genetic parameters

Genetic parameters and their standard errors (SE) are in Table 2 for the three traits and the two breeds. Heritabilities (on the diagonal, Table 2) covered a broad range of values from 0.11 to 0.64. Regardless of the breed, MB had a lower heritability (0.11 to 0.13) than MY (0.33 to 0.45), and MY had a lower heritability than US (0.47 to 0.64). While the heritability of MB was very similar between the Blonde d’Aquitaine and Limousin breeds, the heritability of MY and US was higher in the Limousin (0.45 and 0.64, respectively) than in the Blonde d’Aquitaine breed (0.33 and 0.47, respectively). Regarding the low heritability of MB, two very different estimates are found in the literature, i.e. Phocas et al. [23] estimated a heritability of 0.36 (SE = 0.06) in an earlier study based on the progeny-testing records for the French Limousin breed and Vallée et al. [24] estimated an extremely low heritability of 0.02 (SE = 0.01) for the same trait recorded on-farm in a population of French Charolais cows. Udder morphology has not been extensively studied in beef cows because it is not an easy trait to score, unlike in dairy cows that visit the milking parlor. Our results based on station data led to high heritabilities for US in both breeds. Previous studies reported lower heritabilities based on field records of beef cows: 0.12 (SE = 0.02), 0.20 (SE = 0.04), 0.23 (SE = 0.05) and 0.43 (SE = 0.03), respectively for udder development in Asturiana de los Valles [25], udder volume in Charolais [24], udder score in Hereford [26] and udder width in the Rendena dual-purpose breed [27]. Heritability estimates for MY were moderate and considerably higher than those for maternal weaning weight: 0.13 in the Blonde d’Aquitaine and 0.12 in the Limousin breed, based on farm records [28]. Although maternal effects on calf growth are generally expected to be due to the dam’s milk production [29], a maternal effect on weaning weight is a more complex trait than milk production and accounts for milk quality, maternal bonding and calf behavior. In addition, assessing phenotypes on a station rather than on a farm can markedly limit any environmental effects and therefore provide higher heritability estimates than those based on field data. Using a similar measurement of milk production as that used in our study, MacNeil and Mott [26] reported a heritability of 0.25 (SE = 0.06) for MY in Hereford cattle.

**Table 2:** Genetic parameters for the three traits in Blonde d’Aquitaine and Limousin cattle

	Blonde d’Aquitaine			Limousin		
Trait	US	MB	MY	US	MB	MY
US	0.470 (0.099)	0.312 (0.240)	0.572 (0.151)	0.640 (0.121)	0.790 (0.228)	0.994 (0.072)
MB	*0.042*	0.135 (0.067)	0.584 (0.244)	*0.194*	0.108 (0.068)	0.720 (0.260)
MY	*0.281*	*0.163*	0.331 (0.092)	*0.305*	*0.161*	0.455 (0.113)

Heritabilities are underlined on the diagonal, genetic correlations are above the diagonal with standard errors in brackets, phenotypic correlations are in italics below the diagonal (standard errors ranging from 0.03 to 0.04)

*MB* maternal behavior, *US* udder score, *MY* milk yield

The phenotypic correlations (Table 2) were positive between the three traits in both breeds. The strongest phenotypic correlations were around 0.30 between MY and US in both breeds. Genetic correlations (Table 2) were generally stronger. Genetic correlations between the three traits ranged from 0.31 to 0.58 in the Blonde d’Aquitaine breed and from 0.72 to 0.99 in the Limousin breed. Due to the small number of records, SE of the genetic correlations were relatively large, ranging from 0.07 to 0.26.

The environmental correlations were all less than 0.10 in absolute value, except between US and MY in the Limousin breed with a value of −0.52. Persson Waller et al. [30] reported that beef cows with pendulous udders were at risk of subclinical mastitis. Marked swelling of the udder may then induce mastitis and consequently reduce milk production. This phenomenon may explain the strong negative environmental correlation between US and MY.

The three traits were clearly more strongly genetically correlated in the Limousin than in the Blonde d’Aquitaine breed. In particular, US and MY phenotypes are expected to be controlled by the same pools of genes in the Limousin cows, which may not be the case for the Blonde d’Aquitaine cow for which only a moderate genetic association (0.57, SE = 0.15) was estimated. MacNeil and Mott [26] estimated a moderately positive genetic correlation (0.36, SE = 0.16) between udder score and milk production in Hereford cattle, while Berry et al. [31] also derived positive but moderate correlations (ranging from 0.32 to 0.48) between MY and some udder traits (fore-udder attachment, udder support and rear-udder height) in Holstein primiparous cows.

### Detection of QTL within breed

Regardless of the breed, very few QTL were detected for MB, which is probably due to its low heritability compared to the moderate to high heritabilities estimated for US and MY. Thirty to 56 QTL were detected for US and MY with a threshold logBF level equal to 8.

A strong correlation (0.98) between the estimates of ${\text{P}}_{\text{i}}$ (underlying the BF) and the absolute value of the estimated effects for each SNP was computed for all traits. Therefore, a QTL that is supported by strong evidence based on the BF value is expected to be a QTL with an important effect on performance.

#### QTL detected for the Blonde d’Aquitaine breed

In the Blonde d’Aquitaine population, 56, 53 and two QTL were detected for US, MY and MB, respectively. The main QTL region for MB was on chromosome 6 (Table 3; Fig. 1) and it explained 18.8 % of the estimated total accuracy of genomic prediction for MB (r = 0.72). The *NPY1R* and *NPY5R* genes (Table 6) are located close to the peak SNP of this QTL and represent good candidate genes. Longo et al. [32] showed that these two genes were co-expressed and linked to stress and anxiety in mice. In addition, Muroi and Ishii [33] demonstrated the relationship between *NPY1R* and maternal behavior by assessing mice that crouch over their pups after a period of separation. The second QTL for MB was located on chromosome 26 (Table 3) and explained 21.5 % of the accuracy of genomic prediction. The pseudogene *ADRA2A* (Table 6) present in the corresponding human, murine and rat genomic regions that align with the QTL region detected here, encodes an adrenergic receptor which is related to several behavior traits [34], and thus, it is a likely candidate gene for MB.

**Table 3:** QTL regions (in Mb) with strong evidence for the peak SNP and corresponding candidate genes in the Blonde d’Aquitaine breed

Trait	Chr^a^	Start–end positions (in Mb)	Peak position (Mb)	Peak logBF	Contribution to r^b^ (%)	Candidate gene
MB	6	2.362–2.974	2.486743	8.3	18.8	*NPY1R*, *NPY5R*
MB	26	31.569–32.67	32.099795	8.2	21.5	*ADRA2A*
US	6	88.485–88.959	88.922396	16.3	42.5	*GC*
MY	5	28.577–29.137	29.072132	13.4	15.2	*SLC11A2*
MY	6	88.485–89.223	88.919352	14.4	20.6	*GC*
MY	10	69.747–72.705	70.306697	12.8	20.8	–
MY	13	82.728–84.013	83.805618	12.1	14.7	*DOK5*
MY	20	3.861–7.327	5.504819	13.2	19.3	–
MY	27	42.375–43.266	42.896895	13.4	15.9	*UBE2E2*

*MB* maternal behavior, *US* udder score, *MY* milk yield

^a^Chromosome

^b^r is the accuracy of the genomic prediction for the reference population

**Fig. 2:**
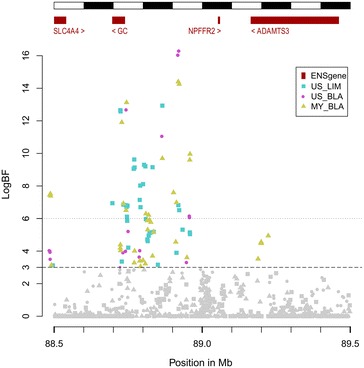
Plot of logBF for SNPs in a 1-Mb QTL region on chromosome 6 for udder score and milk yield. The logBF values for all SNPs were plotted between 88.5 and 89.5 Mb on chromosome 6 for the detection of QTL for milk yield (MY_BLA) and udder score (US_BLA) in Blonde d’Aquitaine and Limousin (US_LIM) cows. The locations of known genes (ENSgene) within the region are indicated at the *top of the figure*

Only one QTL with strong evidence was detected for US and was located on chromosome 6 (Table 3). Its peak logBF value (16.3) was the highest among all the QTL detected during this analysis. This QTL explained 42.5 % of the estimated total accuracy of genomic prediction for US (r = 0.81). This QTL region was also detected with strong evidence for MY in the Blonde d’Aquitaine population (Fig. 1) and explained 20.6 % of the estimated total accuracy of genomic prediction for MY (r = 0.85). The peak SNPs for these two QTL were separated by a distance of 0.003 Mb on the HD chip, which indicates that they are probably associated with the same causal mutation that has a pleiotropic effect on US and MY. Goddard et al. [35] also detected this region for milk yield in the Holstein breed based on bull sequence data. The region that contains the peak SNP is intergenic but close (<0.2 Mb) to the *GC* gene (Table 6), which encodes the vitamin D binding protein (Fig. 2). This gene, or its regulatory sequence, can be considered as a good candidate because vitamin D is involved in blood calcium homeostasis, which is required for stable milk production. As shown by Horst et al. [36], a deregulation of calcium homeostasis reduces milk production and can lead to milk fever.

In addition to this pleiotropic QTL on chromosome 6, five other QTL with strong evidence were detected for MY in the Blonde d’Aquitaine breed and explained 15.2 to 20.8 % of the accuracy of genomic prediction. However, combined together, the six QTL regions only explained 31.0 % of the estimated total accuracy of genomic prediction for MY.

On chromosome 5, three sequence alignments of the *SLC11A2* gene (Table 6) were found in the vicinity of the peak SNP (29.072 Mb) of a QTL with strong evidence for MY (Table 3). The *SLC11A2* gene is a likely candidate for this QTL because it encodes a divalent metal transporter protein that is involved in regulating essential nutrients such as iron in the milk [37].

The *DOK5* gene (Table 6) which was the only gene identified in the QTL region that was detected with strong evidence for MY on chromosome 13. *DOK5* encodes a member of the DOK family of membrane proteins, which are adapter proteins involved in signal transduction, and DOK5 has a potential role in insulin and IGF-1 action [38]. This gene can be considered as a candidate gene because IGF-1 stimulates protein synthesis in bovine mammary epithelial cells [39].

On chromosome 27, a QTL with strong evidence for MY (Table 3) corresponded to a single gene that aligned with mouse, rat and sheep sequences: *UBE2E2* (Table 6). This gene is a good candidate for MY because it encodes a protein that plays an important role in the synthesis and secretion of insulin and may influence fat and protein content in milk [40, 41].

Two large regions with no particular identified genes were detected with strong evidence for MY on chromosomes 10 and 20 (Table 3). The QTL on chromosome 20 was also detected for maternal weaning weight based on Blonde d’Aquitaine field records (unpublished results).

#### QTL detected for the Limousin breed

For the Limousin breed, 54, 30 and one QTL were detected for US, MB and MY, respectively. The only QTL detected for MB in the Limousin breed was on chromosome 3 (Table 4) and explained 24.6 % of the estimated total accuracy of genomic prediction for MB (r = 0.75). No particular candidate gene was identified in the region that might play a role in MB (Fig. 1).

**Table 4:** QTL regions with strong evidence for the peak SNP and corresponding candidate genes in the Limousin breed

Trait	Chr^a^	Start–end position (in Mb)	Peak position (Mb)	Peak logBF	Contribution to r^b^ (%)	Candidate gene
MB	3	66.755–68.481	67.907266	11.5	24.6	–
US	2	128.497–129.297	128.921997	12.2	16.5	–
US	6	67.175–69.578	68.004090	12.5	28.8	*CORIN*
US	6	87.542–88.959	88.865430	12.9	20.6	*GC*
US	8	100.655–102.859	101.161417	12	19.5	*PALM2*
US	19	30.26–30.315	30.259765	13.9	19.4	*SCO1*
US	29	33.826–35.441	34.797963	12.9	23.9	–
MY	5	104.195–105.769	104.616203	12.8	23.2	–
MY	15	43.616–45.452	44.477700	14.4	20.1	*RPL27A*

*MB* maternal behavior, *US* udder score, *MY* milk yield

^a^Chromosome

^b^r is the accuracy of the genomic prediction for the reference population

Six QTL were detected with strong evidence for US in the Limousin breed and explained 16.5 to 28.8 % of the accuracy of genomic prediction. Combined together, these six QTL explained 37.4 % of the estimated total accuracy of genomic prediction for US (r = 0.87). The QTL on chromosome 6 had already been reported for MY and US in the Blonde d’Aquitaine breed (Fig. 2). As discussed above, it was the only QTL detected with strong evidence in both breeds. It explained 20.6 % of the accuracy of genomic prediction for US in the Limousin breed. A second QTL was detected on chromosome 6 with strong evidence (Table 4). The peak SNP was within the *CORIN* gene (Table 6) which plays various roles in the regulation of blood pressure. The *CORIN* gene is up-regulated in the decidua of the pregnant uterus, which suggests a potential role for CORIN during pregnancy [42].

Another QTL with strong evidence was detected on chromosome 19 (Table 4). This QTL contained the *SCO1* gene (Table 6), which encodes a protein involved in the mitochondrial respiratory chain that is essential for adenosine triphosphate (ATP) synthesis [43]. Because of the possible link between this metabolic pathway and udder development, *SCO1* is a putative candidate gene. Concerning the QTL with strong evidence on chromosome 8 (Table 4), the *PALM2* gene (Table 6), which is involved in the process of cell formation [44], was located near the peak SNP within the QTL region. Finally, two QTL with strong evidence for US but without any particular candidate genes were detected on chromosomes 2 and 29 (Table 4).

Two QTL were detected with strong evidence for MY on chromosomes 5 and 15 in the Limousin breed (Table 4). We did not identify candidate genes in these two QTL regions, although they explained 23.2 and 20.1 % of the accuracy of genomic prediction. Sukegawa et al. [45] reported a significant association between marbling in Japanese Black cattle and the *RPL27A* gene (Table 6), located next to the peak of the QTL of chromosome 15. Because marbling is related to the metabolic pathway of fat, a possible link may exist between this gene and MY.

#### QTL shared by genetically-correlated traits within each breed

In this study, the three traits that were considered to be linked to maternal care and suckling performance were moderately to strongly correlated within breeds. Therefore, we assumed that a set of common QTL with a role in at least two of the three traits under study would be found. However, this was not the case between MB and either of the other two traits. The absence of common QTL might be related to the very small number of QTL that were detected for MB in the Limousin and Blonde d’Aquitaine breeds.

Four of the QTL regions that were identified for the Blonde d’Aquitaine breed were common to those detected for US and MY (Table 5). Combined together, these four QTL explained 44.4 % of the estimated total accuracy of genomic prediction for US (r = 0.81) and 22.9 % of the estimated total accuracy of genomic prediction for MY (r = 0.85). As previously noted, the only QTL that was detected with strong evidence for US on chromosome 6 was one of these four common QTL. The contributions of this QTL to the accuracies of genomic predictions for US and MY (42.5 % for US and 20.6 % for MY) were nearly the same as the overall contributions of the four QTL combined together (44.4 % for US and 22.9 % for MY). Thus, the positive genetic correlation that was found between US and MY (Table 2) seems to be mainly explained by this single QTL region on chromosome 6, probably linked to *GC* gene variants in the Blonde d’Aquitaine breed. Analysis of the three common regions that were detected with a lower logBF peak led to the identification of another putative candidate gene that affects milk production (Fig. 1). On chromosome 8, the *RGP1* gene (Table 6) was identified in the vicinity of the peak SNP (at 0.02 Mb) within the QTL for MY (Table 5). This gene is involved in the conversion of guanosine diphosphate (GDP) into guanosine triphosphate (GTP) and is linked to milk production [46].

**Table 5:** Common QTL regions between traits and/or breeds and corresponding candidate genes

Trait 1–trait 2	Chr^a^	Common region (Mb)	Peak position trait 1	Peak position trait 2	Candidate gene
US_BLA–MY_BLA	4	44.177–44.917	44.260073	44.198598	
US_BLA–MY_BLA	6	88.485–88.959	88.922396	88.919352	*GC*
US_BLA–MY_BLA	8	60.348–60.353	61.044151	60.352572	*RGP1*
US_BLA–MY_BLA	28	43.511–44.63	43.242413	44.036312	
US_LIM–MY_LIM	1	106.449–106.515	106.73685	106.008757	*OTOL1*
US_LIM–MY_LIM	6	68.405–68.664	68.00409	68.488326	*CORIN*
US_BLA–US_LIM	6	52.894–52.978	52.814243	52.897667	
US_BLA–US_LIM	6	63.663–65.139	65.495791	63.440877	
US_BLA–US_LIM	6	88.485–88.959	88.922396	88.86543	*GC*
US_BLA–US_LIM	14	65.751–65.862	65.806497	65.802602	*PABPC1*
US_BLA–US_LIM	29	37.426–37.505	37.459691	40.655558	
MY_BLA–MY_LIM	3	27.53–28.035	27.842537	27.657684	*CASQ2*

*MB* maternal behavior, *US* udder score, *MY* milk yield

^a^Chromosome

**Table 6:** Symbol, name and position of candidate genes

Gene symbol	Gene name	Chr^a^	Position (Mb)
*OTOL1*	*Otolin 1*	1	106.700432–106.731202
*CASQ2*	*Calsequestrin 2*	3	27.658862–27.729416
*SLC11A2*	*Solute Carrier Family 11 Member 2*	5	28.887967–28.914607
*NPY5R*	*Neuropeptide Y Receptor Type 5*	6	2.385668–2.396509
*NPY1R*	*Neuropeptide Y Receptor Type 1*	6	2.415744–2.423445
*CORIN*	*Corin Serine Peptidase*	6	67.925293–68.176596
*GC*	*group*-*specific component*	6	88.695940–88.739180
*RGP1*	*Retrograde Golgi Transport Homolog*	8	60.328033–60.333812
*PALM2*	*Paralemmin 2*	8	101.018440–101.182488
*DOK5*	*Docking Protein 5*	13	82.696138–82.803966
*PABPC1*	*Poly(A) Binding Protein*, *Cytoplasmic 1*	14	65.816006–65.833756
*RPL27A*	*Ribosomal Protein L27a*	15	44.469327–44.472127
*SCO1*	*Cytochrome C Oxidase Assembly Protein*	19	30.276156–30.296404
*ADRA2A*	*Adrenoceptor Alpha 2A*	26	31.870225–31.871586
*UBE2E2*	*Ubiquitin*-*Conjugating Enzyme E2E 2*	27	42.558023–42.569249

^a^Chromosome

Only two common QTL were detected for US and MY in the Limousin breed. Combined together, these two QTL regions explained 31.6 % of the estimated total accuracy of genomic prediction for US (r = 0.87) and 22.6 % of the estimated total accuracy of genomic prediction for MY (r = 0.84). The QTL region detected on chromosome 1 (Table 5) had a peak SNP that was the same for the two traits. The peak SNP was located in the *OTOL1* gene (Table 6) that has variants that affect body mass index in human Japanese or Korean populations [47]. This gene appears to play a role in the energy storage process, and, thus, may impact milk production.

Nevertheless, these two QTL alone, which each accounted only for a limited contribution to the accuracy of genomic predictions, cannot explain the very strong genetic correlation between MY and US. To investigate further this strong genetic correlation, we estimated the contributions of all the SNPs present in the two QTL or 20 putative QTL regions that were common to US and MY, and they accounted for 55.5 and 59.9 % of the accuracy of genomic predictions for US and MY, respectively. These contributions were a little larger than those estimated for the Blonde d’Aquitaine breed (52.8 and 46.1 % for US and MY, respectively), but could not explain the large discrepancy between the genetic correlations in the Limousin and the Blonde d’Aquitaine populations. As a consequence, we hypothesize that a very large number of small polygenic effects explain the very strong correlation observed between US and MY in the Limousin breed. In addition, our results indicate that the sources of additive genetic variance of US and MY clearly differ in the Limousin and Blonde d’Aquitaine breeds.

#### QTL shared by the two breeds for the same trait

Blonde d’Aquitaine and Limousin are genetically close breeds because of their common geographical origin in south-western France. By studying the genetic history of 47 cattle breeds, Gautier et al. [7] showed that Limousin was the closest breed to Blonde d’Aquitaine based on the F_ST_ approach using 50K SNP genotyping data. Servin et al. [48] confirmed these results using 700K SNP genotyping data. Therefore, we assumed that a large set of common QTL for the same trait would be found across the two breeds. Indeed, during the second part of the twentieth century, selective breeding was essentially focused on improving growth and conformation traits rather than maternal traits, thus little divergence was expected to have affected the sources of additive genetic variance of maternal traits between the two breeds. However, our results on the within-breed detection of QTL strongly questioned this initial assumption.

First, we found no common QTL for MB across the two breeds. Second, our results provided strong evidence (logBF > 8) for only one QTL for MY, which was shared by the two breeds. This QTL was located on chromosome 3 (Table 5) and explained only 16.5 and 18.5 % of the accuracy of genomic predictions for the Blonde d’Aquitaine and Limousin breeds, respectively. The peak SNP for MY in the Limousin dataset was located 0.001 Mb upstream of the *CASQ2* gene (Table 6). This gene encodes a calcium binding protein that stores calcium for muscle function [49]. In dairy cows, Horst et al. [36] demonstrated the relationship between calcium storage and MY, which makes *CASQ2* a possible candidate gene. However, this common QTL was not the major source of genetic variation in MY for the two breeds. If all common (1) and putative QTL (17) for MY are considered, only 43.8 and 49.5 % of the accuracy of genomic predictions was explained for Blonde d’Aquitaine and Limousin, respectively.

Among the 55 QTL detected for US with strong evidence within breeds, five were shared by the two breeds. Combined together, these five QTL explained 44.0 and 34.0 % of the accuracy of genomic predictions for US in the Blonde d’Aquitaine and Limousin breeds, respectively; if the 33 putative common QTL were added, contributions of about 54 % to the genomic prediction accuracies were estimated for each breed.

The QTL for US on chromosome 6 that was supported by strong evidence in both breeds contributed substantially to the accuracy of genomic prediction, although the proportion was smaller for the Limousin (20.6 %) than for the Blonde d’Aquitaine (42.5 %) breed. Detecting this QTL in both breeds further supported the *GC* gene as a major candidate gene for US in beef cattle. No candidate gene related to US was identified for three of the four other QTL shared by the two breeds. Only one candidate gene was proposed for the common QTL located on chromosome 14 (Table 5). The two peak SNPs were very close to each other (0.004 Mb) and were located about 0.01 Mb from the *PABPC1* gene (Table 6), which regulates multiple aspects of mRNA translation and stability [50].

## Conclusions

Apart from maternal behavior, which had a low heritability in both breeds (0.11 to 0.13), heritabilities for the two suckling traits, MY and US, ranged from moderate values (0.33 to 0.47) in the Blonde d’Aquitaine to high values (0.45 to 0.64) in the Limousin breed. Genetic correlations between these three traits were moderately positive (0.31 to 0.58) in the Blonde d’Aquitaine cows, but strongly positive (0.72 to 0.99) in the Limousin cows. These results allow for the first time to evaluate the major sources of additive genetic variation of maternal traits in both breeds. They indicate that the polymorphisms of interest for selective breeding may differ substantially between these two breeds although the genetic distance between them is short compared to bovine populations as a whole. Regarding MB, strong evidence supported two QTL in the Blonde d’Aquitaine and one QTL in the Limousin breed and we identified two major candidate genes i.e. *NPY1R* and *ADRA2A*. Regarding MY, 30 and 53 QTL were detected for the Limousin and Blonde d’Aquitaine breeds, respectively; of these, only two (Limousin) and six (Blonde d’Aquitaine) were supported by strong evidence. Several good candidate genes were identified for four of the main QTL for MY in the Blonde d’Aquitaine breed: in particular, the upstream region close to the *CG* gene, but also *SLC11A2*, *DOK5* and *UBE2E2*. However, no convincing candidate genes were found for the two main QTL for MY in the Limousin breed. In addition, only one QTL region for MY was shared by the two breeds and we did not identify any likely candidate gene to explain this common effect.

For US, 54 and 56 QTL were detected in the Limousin and Blonde d’Aquitaine breeds, respectively. The same QTL with the strongest evidence was found for the Blonde d’Aquitaine and Limousin breeds and it was located next to the *CG* gene, which was also identified for MY in the Blonde d’Aquitaine breed. Four other common QTL were involved in udder swelling in both breeds. However, apart from the *CG* gene, only the *PABPC1* gene was identified as a putative candidate gene underlying the common QTL. These common QTL between the Limousin and Blonde d’Aquitaine breeds that are involved in the genetic variation of US were the only clear evidence that agreed with their genetic proximity.

Very few pleiotropic QTL were detected for both US and MY. In Blonde d’Aquitaine, the genetic correlation between the two traits was largely due to the QTL located near the *CG* gene and that had a strong effect on both US and MY. Genotyping data for the Limousin breed did not allow us to clarify the very high genetic correlation between MY and US that was estimated based on performance and pedigree information. As a consequence, we assume that numerous pleiotropic regions with minor effects must explain the polygenic correlation, without any statistical possibility of detecting the corresponding QTL because of the limited size of our dataset.

Nevertheless, we identified 15 candidate genes that could explain the major sources of additive genetic variation in maternal traits in continental beef breeds. Among these, four genes may have a role in the significant variations in suckling performance of both Limousin and Blonde d’Aquitaine cows. Future studies based on the analysis of sequence data for the highlighted regions may allow us to determine the causal mutations.

## Additional file

10.1186/s12711-016-0223-z Description of the recorded traits. Description: Description of the way the three traits were recorded on a station.
