# Supplementary material for: Insights into the genetic variation of maternal behavior and suckling performance of continental beef cows
Source: Genet Sel Evol. 2016 Jun 22;48:45. doi: 10.1186/s12711-016-0223-z (PMC4918023; doi:10.1186/s12711-016-0223-z)
Supplement: Supplementary file 1 — 10.1186/s12711-016-0223-z Description of the recorded traits. Description: Description of the way the three traits were recorded on a station. [file 12711_2016_223_MOESM1_ESM.pdf]

Additional file 1
Description of the recorded traits

All the traits were recorded in a single progeny test station per breed. Daughters of the test sires enter the progeny test station of their breed selection unit as weaned heifers. Heifers, born within a restricted season, are bought in commercial herds to be a representative sample of the purebred daughters of the AI candidate sires. Measures are recorded from the weaning of the heifers to the weaning of their calves. Heifers are inseminated during a fixed 10-weeks period in order to get a first calving at 2 years of age. Only two purebred mating bulls are used each year. They are used for several years in order to correct for their effect on calf performance and on female fertility and calving ease.

- **Udder swelling score (US)**

The udder swelling score (US) assess the udder swelling before calving with a five-point scale ranging from 1 for the least swollen udder to 5 for the most swollen udder. It is scored approximately one week before calving by the same technician for all the contemporary heifers. The teat size is not taken into account in this score.
Score 1 corresponds to a very tight attachment of the udder, associated to a very pronounced median suspensory ligament; score 2 corresponds to a tight attachment of the udder, associated to a pronounced median suspensory ligament; score 3 corresponds to an intermediate attachment of the udder; score 4 corresponds to a loose attachment of the udder, associated to a weak median suspensory ligament; and score 5 corresponds to a very loose attachment of the udder, associated to a very weak median suspensory ligament.

- **Maternal behavior (MB)**

The maternal behavior (MB) describes the intensity of the dam's protective behavior towards her calf. It is scored during the first hour after parturition.
Score 1 corresponds to a dam who did not pay attention to her calf; score 3 corresponds to a dam taking care of her calf, but only after a little while; score 5 corresponds to a dam who actively stimulated the newborn calf to suck, particularly by licking it immediately after calving.
The scores 2 and 4 are intermediate between the previously described scores.

Within a year of performance recording of the same contemporary group of cows, three technicians participate to the scoring of MB. The scorer is not registered in the database. In consequence, no scorer effect can be fitted in the genetic evaluation model. However, the scores are expected to be consistent across scorers because the scorers follow precise guidelines for scoring and they are used to standardize their notations each year by scoring together a few tens calving.

- **Milk yield (MY)**

Milk yield (MY) is assessed using the calf weigh-suckle-weigh technique to obtain an estimate of suckling performance. At two test days (two months after calving M2, and four months after calving M4), the calf is separated from its dam during 24h, starting in the evening of the day before the test day.

Within a test day, two limited time periods of 15 minutes of suckling are managed by the technicians: one in the morning and the other in the evening. Each time, the calf is weighted before and after suckling to assess the amount of milk suckled. These two weights are summed to obtain a daily milk production. In the case of a dry cow, the milk weight is 0. The final trait MY is estimated considering the weighted mean of the two test day controls: MY = (M2 + 2 M4) / 3.
